# The Behavior of Gangue During the Flotation of a Sulfidic PGM-Bearing Ore in Response to Various Monovalent and Divalent Ions in Process Water

**DOI:** 10.3389/fchem.2020.00079

**Published:** 2020-03-18

**Authors:** Malibongwe Shadrach Manono, Kirsten Claire Corin, Jennifer Gael Wiese

**Affiliations:** Centre for Minerals Research, Department of Chemical Engineering, University of Cape Town, Cape Town, South Africa

**Keywords:** flotation, gangue, inorganic electrolytes, ions, sulfides, talc, water

## Abstract

Mineral concentrators are becoming increasingly aware of the importance of the quality of the water that they feed into their milling and flotation circuits. It is speculated that different inorganic constituents of process water may yield different flotation results owing to the electrolyte–reagent–mineral interactions occurring in the pulp phase. These interactions are said to be specific to ion type, reagent type, and mineral or ore type. It therefore stands to reason that there is a need to develop an understanding of the specific ion effects on both the pulp phase and the froth phase phenomena, such that the chemistry and the quality of process water can be monitored and controlled in a manner that does not negatively affect the flotation performance. Previous research has shown that inorganic electrolytes may impact the hydrophobicity and the floatability of mineral particles and could in turn affect froth stability, entrainment, and thus mineral grades and recoveries. In this study, the floatability of a Cu-Ni-PGM-bearing Merensky ore is tested on a bench-scale flotation system in various single salt solutions, *viz*., CaCl_2_, CaSO_4_, Ca(NO_3_)_2_, MgCl_2_, Mg(NO_3_)_2_, MgSO_4_, NaCl, NaNO_3_, and Na_2_SO_4_, in order to examine specific ion effects on gangue recovery. Coagulation and zeta potential tests are conducted in order to establish the nature of the impact that specific ions have on the behavior of gangue in flotation. The findings of this work have shown that single salt solutions containing ${\text{NO}}_{3}^{-}$ ions resulted in a strong depression of gangue compared to those solutions containing Cl^−^ and SO_4_^2−^ ions. It was also shown that the divalent Ca^2+^ and Mg^2+^ showed a stronger depression of gangue compared to the monovalent Na^+^. Ca^2+^, in comparison to Na^+^, resulted in an increase in the coagulation of the ore as well as an increase in the zeta potential of talc. Overall, the findings of this paper suggest that the presence of Ca^2+^ and Mg^2+^ in process water would most likely create conditions that promote gangue depression.

## Research Key Highlights

- Solutions containing ${\text{NO}}_{3}^{-}$ and Ca^2+^ increased the depression of gangue as shown by lower gangue recoveries.
- The concentrate grades were higher in solutions containing Ca^2+^ and ${\text{NO}}_{3}^{-}$ compared to those which contained Na^+^, Cl^−^, and SO_4_^2−^.
- Ca^2+^ and ${\text{NO}}_{3}^{-}$ resulted in a less negative zeta potential compared to Na^+^ and SO_4_^2−^.
- Coagulation was enhanced in Ca^2+^- and ${\text{NO}}_{3}^{-}$-containing solutions.
- Ca^2+^-containing process waters could be beneficial for floatable gangue depression.

## Introduction

It is acknowledged that water should be considered as a reagent in flotation and, as such, the quality of process water becomes an important factor to consider as it may alter the pulp chemistry and thereby affect the flotation (Corin et al., 2011; Corin and Wiese, 2014). Previous work considered specific ions present in process water and their effects on the flotation of a Cu-Ni-PGM ore (Manono et al., 2016). Much of the conclusions made from Manono et al. (2016) gave rise to speculations of ions, being there in process water, which promoted gangue depression better than others. However, there was no fundamental evidence to support the speculation of an enhancement of gangue depression. Thus, the purpose of this study is to ascertain whether there are specific ion effects on gangue depression. As the separation of value-bearing minerals from the non-value-bearing gangue minerals is the core and fundamental purpose of flotation, the depression of gangue as a critical aspect of flotation is important to consider. Rao and Finch (1989) demonstrated that recycled water may negatively affect mineral recoveries and grades owing to an accumulation of inorganic and organic substances which alter the pulp chemistry and thereby affect the flotation. A study conducted by Muzenda (2010) showed that recycled process water containing Cu^2+^, Pb^2+^, and Fe^2+^ had adverse effects on the flotation of a PGM ore from the UG2 reef of the Bushveld Igneous Complex of South Africa in that there occurred an inadvertent activation of non-sulfide gangue which decreased the concentrate grades. This could be explained by a more recent study by Liu et al. (2014) which suggested that some electrolytes in process water could inhibit the adsorption of xanthates on sulfide minerals due to competition for adsorption onto the mineral surface. Also, Castro et al. (2013) showed that the flotation of molybdenite in the presence of Mg^2+^ ions resulted in molybdenite depression due to the presence of magnesium hydroxyl complexes and colloidal magnesium hydroxide coatings on molybdenite particles, rendering them hydrophilic. Laskowski et al. (2007) also showed that the presence of high Ca^2+^ concentration in process water improved the adsorption of CMC onto talc through an acid–base interaction and thus promoted the depression of talc. Biçak et al. (2012) concluded that dissolved metal ions and sulfide ions mainly in the form of SO_4_^2−^ and S_2_O_3_^2−^ alter the surface chemistry of minerals in the pulp phase. Ikumapayi et al. (2012) also showed that galena was more passivated in Ca^2+^ and SO_4_^2−^ and, as a result, mineral recoveries were reduced. Shackleton et al. (2012) investigated the water quality effects on moncheite–pyroxene and pentlandite–pyroxene composites and proposed that ions found in process waters passivated the mineral surfaces and inhibited the collector adsorption, thereby reducing the floatability of moncheite, pentlandite, and pyroxene. Their findings also suggested that the specific ions in the process water played a far more significant role compared to the overall ionic strength of the process water. This therefore forms the basis of the work carried out in the study presented in this paper. It is evident, from the literature presented, that fundamental, robust, and more comprehensive studies on the effects of specific inorganic electrolytes on the floatability of sulfidic PGM ores are needed. Knowledge of the influence of each specific process water constituent on flotation is vital as it would potentially have both technical and financial benefits in that costs could be saved on wastewater treatment if research proves that some flotation circuits could still perform at their best in recycled process water containing constituents such as inorganic electrolytes which would have been removed due to limited knowledge of their specific impact on flotation performance. Such in-depth research knowledge is crucial in the development and implementation of closed water circuits in mineral concentrators. It is anticipated that understanding the impact of different process water constituents, leading to a successful implementation of closed water circuits, will contribute toward the sustainability of mineral concentrators as a result of minimal or zero process water discharge to the environment, minimal make-up water requirement, a reduction in process water treatment before reuse, savings on treatment costs, and a reduction in fresh reagent dosing. Thus, the aim of this study is to ascertain whether there are ions in flotation with dominant effects on the gangue depression of a Merensky ore. Talc is used as proxy for the naturally floatable gangue common in Merensky ores.

## Materials and Methods

### Synthetic Plant Water and Single Salt Quality

Synthetic plant water (hereafter referred to as 1SPW or 1Plant) and various single salt solutions of the ionic strength and the chemical compositions shown in Table 1 were prepared. These aqueous synthetic plant water and single salt solutions were made from distilled water and inorganic salts to ensure that the concentration of the water contained the required total dissolved solids and ionic strength as shown in Table 1.

**Table 1:** Chemical compositions of synthetic plant water and the various single salt solutions tested in this study.

**Water type**	**Ca^**2+**^ (mg/l)**	**Mg^**2+**^ (mg/l)**	**Na^**+**^ (mg/l)**	**Cl^**−**^ (mg/l)**	**SO_4_^2−^ (mg/l)**	**${\text{NO}}_{3}^{-}$ (mg/l)**	**CO_3_^2−^ (mg/l)**	**Total dissolved solids (mg/l)**	**Ionic strength [M]**
1SPW	80	70	153	287	240	176	17	1,023	0.0213
NaCl	-	-	490	755	-	-	-	1,245	0.0213
Na_2_SO_4_	-	-	326	-	682	-	-	1,009	0.0213
NaNO_3_	-	-	490	-	-	1,321	-	1,810	0.0213
CaCl_2_	285	-	-	503	-	-	-	788	0.0213
CaSO_4_	213	-	-	-	512	-	-	725	0.0213
Ca(NO_3_)_2_	285	-	-	-	-	880	-	1,165	0.0213
MgCl_2_	-	173	-	503	-	-	-	676	0.0213
MgSO_4_	-	129	-	-	512	-	-	641	0.0213
Mg(NO_3_)_2_	-	173		-	-	880	-	1,053	0.0213

### Ore Preparation and Milling

A Cu-Ni-PGM-containing ore from the Merensky reef, within the Bushveld Igneous Complex of South Africa, was used throughout the bench-scale flotation tests. The bulk sample was crushed, riffled, and split into samples weighing 1 kg using a rotary splitter. The 1-kg ore samples were milled in a tumbling mill in the presence of the particular water type under study in order to make a slurry of 66% solids in the mill at the end of milling. Sodium isobutyl xanthate, which was supplied by Senmin, was used as the collector and dosed into the mill at a dosage of 150 g/t. The mill was operated so as to achieve a grind size of 60% passing 75 μm. It is important to note that this grind is typical of industrial concentrator rougher circuits concentrating on Merensky ores. The grinding media was comprised of 20 stainless steel rods with varying diameters: 6 × 25 mm, 8 × 20 mm, 6 × 16 mm. The milled slurry was transferred into a 3-L UCT Barker batch flotation cell immediately. The mineral composition of this ore is shown in Table 2. Pyrrhotite, pentlandite, and chalcopyrite are the main base metal sulfides; these form about 1% of the overall ore feed grade, with the rest being gangue minerals.

**Table 2:** Modal composition: sulfide and gangue minerals present in the ore as determined by QEMSCAN.

**Mineral**	**%**
Pentlandite	0.31
Chalcopyrite	0.25
Pyrrhotite	0.44
Pyrite	0.08
Other sulfides	0.02
Total sulfides	1.09
Plagioclase	43.38
Orthopyroxene	32.60
Olivine	0.59
Clinopyroxene	7.48
Talc	3.51
Serpentine	0.80
Chlorite	0.83
Phlogopite	0.46
Quartz	0.67
Calcite	0.18
Oxides	8.10
Other	0.32
Total	100.00

### Batch Flotation

The standard UCT bench-scale batch flotation procedure was employed on a 3-L Barker cell. The milled slurry was transferred to the cell, and the single salt solution under investigation was added to the cell in order to ensure a pulp density of 35%; thereafter, the impeller was switched on and set to an agitation speed of 1,200 rpm. A syringe was used to draw out a feed sample (which would later be filtered, dried, and weighed for Cu, Ni, and S assays). A polyglycol frother in the form of DOW 200 supplied by Betachem was added to the cell at a dosage of 40 g/t and allowed to condition for 1 min, after which the air supply valve to the cell was opened in order to ensure a constant volumetric air flow rate of 7 L/min. A froth build-up occurred until a constant froth height of 2 cm. Four concentrates were scraped in 15-s intervals and collected at 2, 4, 6, and 10 min into concentrate dishes named C1, C2, C3, and C4, respectively. Once all of the four concentrates were collected, the air supply was switched off; a tails sample was also taken thereafter. The concentrates were weighed in order to account for the amount of water reporting to the concentrate, and thereafter the samples were filtered, dried, weighed, and analyzed for Cu, Ni, and S in the UCT Chemical Engineering Analytical Services Laboratory using XRF and Leco. All tests were performed in duplicates, and the error bars are shown in the batch flotation results. Batch flotation tests were performed at room temperature (25°C).

### Solid Settling Tests

Settling tests were performed for single salt solutions of CaCl_2_, CaSO_4_, Ca(NO_3_)_2_, NaCl, NaNO_3_, and Na_2_SO_4_ at a total ionic strength of 0.0213 mol dm^−3^, with each test performed in duplicate. These salts were carefully selected as they showed the most interesting effects on gangue recovery during the flotation of the selected Cu-Ni-PGM ore. Nine grams of Merensky ore were added to 90 ml of the water under investigation in a 100-ml glass beaker to make a slurry containing 10% solids. The contents of the glass beaker were mixed adequately for 1 min using a magnetic stirrer. The pH of the suspension was adjusted to pH 9 using stock/dilute solutions of NaOH or HCl. The suspension was allowed to mix at 500 rpm for 4 min in order to disperse the mixture immediately after pH adjustment. The slurry was then carefully transferred to a 100-ml graduated cylinder. The graduated cylinder was carefully monitored until a clear supernatant liquid was observed against a clear background. A picture was taken, printed, and stuck next to the working bench to serve as a basis for the clear supernatant liquid for the remaining tests. The time taken to reach equivalent settling was recorded. Settling tests were conducted at room temperature (25°C).

### Zeta Potential Measurements on Talc

A Malvern Zetasizer 4 was used to measure the zeta potential of talc at varying pH values. Talc was crushed using a hammer and pulverized thereafter. This was sieved and screened to 100% passing 25 μm. Single salt solutions of Ca(NO_3_)_2_, CaSO_4_, NaNO_3_, and Na_2_SO_4_ were used as dispersants on the Malvern Zetasizer 4. Mg^2+^ salts were excluded since Ca^2+^ and Mg^2+^ showed similar flotation results. Na^+^ salts were included since these showed different flotation results compared to the two (Ca^2+^ and Mg^2+^) divalent cations. Furthermore, zeta potential measurements on talc, performed in the presence of CaCl_2_, MgCl_2_, and NaCl, are reported in Manono et al. (2019); hence, they are not shown in this paper. For each tested single salt solution [i.e., Ca(NO_3_)_2_, CaSO_4_, NaNO_3_, or Na_2_SO_4_], six aliquots of 25 ml were measured and were adjusted to pH values of 2, 4, 6, 8, 10, and 12 using NaOH and HCl. These were allowed to condition for 20 min. Thereafter, 0.0625 g of talc was added to the 25 ml conditioned dispersant, stirred, and left to stand for 1 min. Two milliliters of the supernatant solution was then pipetted into the capillary tube and placed into the Malvern Zetasizer 4. A calibration time of 2 min was allowed, and each reading was taken three times. This procedure was repeated for all the different dispersants. Zeta potential measurements were conducted at room temperature (25°C).

### Inorganic Electrolyte Speciation

In order to investigate specific ion effects on interactions occurring in the pulp phase of flotation at the solid–water interface, inorganic electrolyte speciation calculations were carried out using the Visual MINTEQ (version 3.1). The Visual MINTEQ is an open-source chemical equilibrium modeling software for the prediction or calculation of ion speciation in water based on thermodynamic equilibrium data (Wang et al., 2016). Only single salt solutions of Ca(NO_3_)_2_, CaSO_4_, NaNO_3_, and Na_2_SO_4_ were subjected to the Visual MINTEQ for ion speciation over a pH range of 2–12. Temperature was set at 25°C.

### Statistical Analyses

Statistical analyses were carried out using the Minitab 18.1 Analysis of Means (ANOM). This method uses the output means (e.g., %S grades) for all conditions and calculates an overall or grand mean. It then compares the single test condition output against the total grand mean calculated from all conditions. This would be such that if an output mean for a specific condition falls below the lower bound of the confidence interval, it would be said that the specific test condition resulted in an output mean significantly lower than the total grand mean, whereas if the output fell outside the upper bound of the 95% confidence interval, that condition is said to have resulted in an output that is significantly higher than the total grand mean. However, if a specific test condition resulted in an output that fell within the confidence interval, that output is said to be comparable with the total grand mean (i.e., there is no discernable statistical difference between that specific test condition's finding and the total grand mean, and therefore it cannot be said that the specific test condition resulted in a lower or higher impact compared to the other tested conditions).

## Results

### The Effect of Single Salts on Total Sulfides, Gangue and Water Recoveries, and Total Sulfide Grades

**Figure 1:**
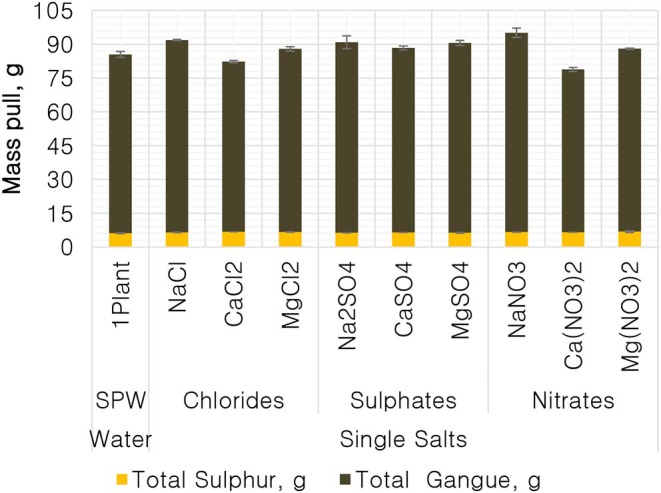
The amount of solids, split into total sulfides and total gangue, reporting to the concentrate for all conditions tested.

**Figure 2:**
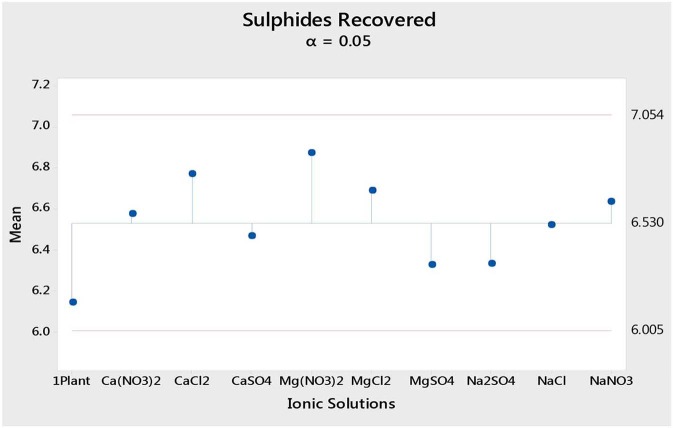
Analysis of means of the amount of sulfides (in grams) reporting to the concentrate for all single salts and synthetic plant water.

**Figure 3:**
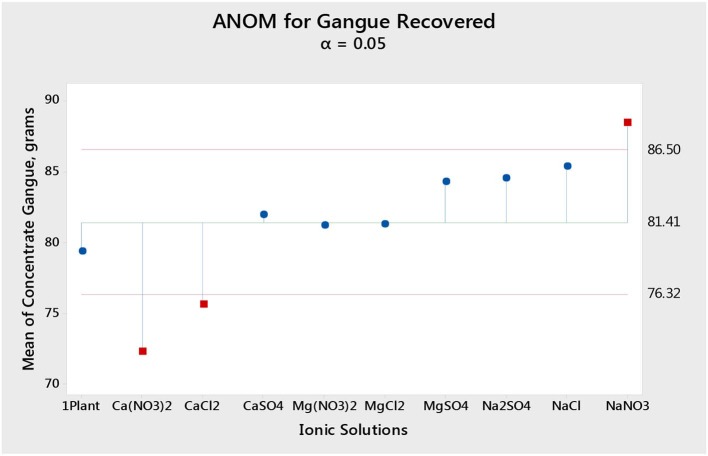
Analysis of means (ANOM) of the amount of gangue reporting to the concentrate for all single salts and synthetic plant water.

Figure 1 shows the total mass pull (i.e., the total amount of solids reporting to the concentrate) for all the inorganic electrolytic solutions. The mass pull is presented as two fractions, namely, the sulfides and the gangue. It is shown that the amount of sulfides reporting to the concentrate did not change with single salt type and the recovered mass of sulfides was the same as that recovered in the presence of synthetic plant water; these results are further supported by the ANOM performed on these data in Figures 2, 3, where it is shown that there is no significant difference between the means of the mass of sulfides obtained with different single salts and of that obtained with synthetic plant water. However, the amount of gangue reporting to the concentrate showed a dependence on the ions present in the single salt solution. Ca(NO_3_)_2_ and CaCl_2_ resulted in a significant decrease in total gangue recovery compared to other single salts, while NaNO_3_ resulted in a significant increase in gangue reporting to the concentrate as shown in Figure 3.

**Figure 4:**
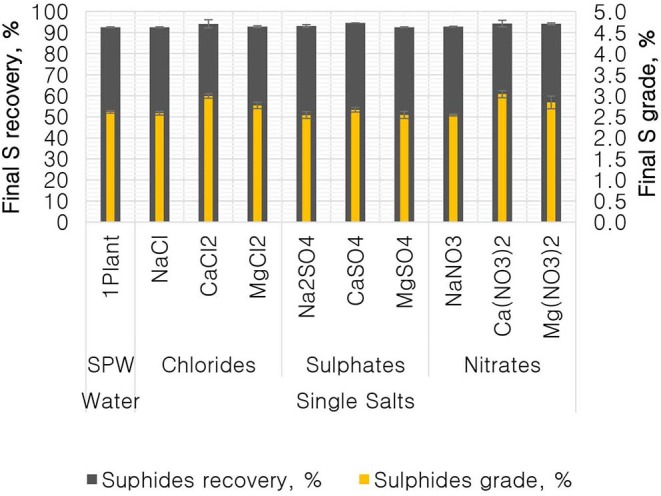
Total sulfide recoveries and grades for all single salts and synthetic plant water.

**Figure 5:**
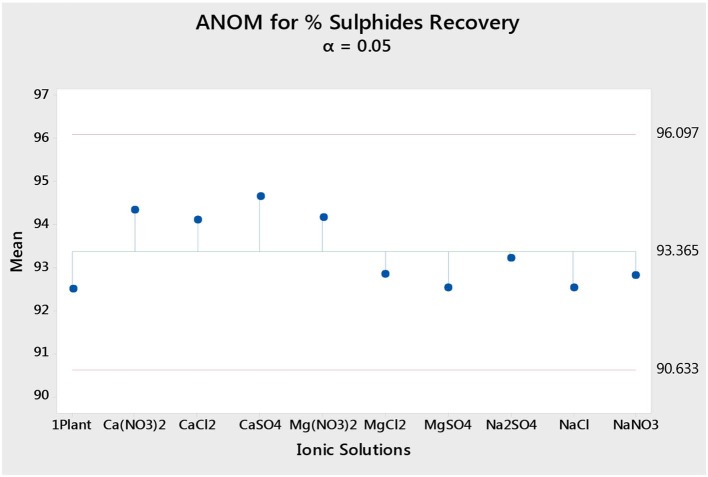
Analysis of means (ANOM) of the total sulfide recovery for all single salts and synthetic plant water.

**Figure 6:**
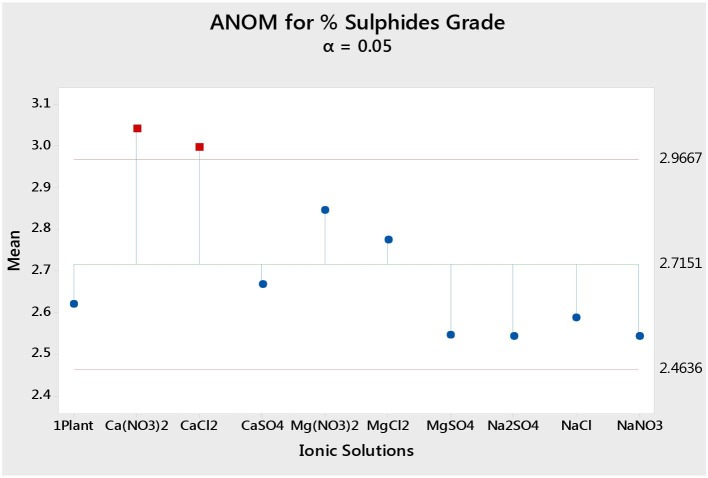
Analysis of means (ANOM) of the total sulfides grade for all single salts and synthetic plant water.

Figure 4 shows the total sulfide recoveries and grades for all tested single salts and synthetic plant water. It is clear that the %S recovery remained unaffected by changes in the inorganic electrolyte solution type, whereas the %S grade proved to be susceptible to changes in the quality of the ionic solution as illustrated in Figures 5, 6. Ca(NO_3_)_2_ and CaCl_2_ resulted in significantly higher concentrate grades compared to all the other single salts as can be seen in Figure 6. It is also interesting to note that the Na^+^-containing single salt solutions resulted in grades slightly lower than the grand mean shown by the ANOM presented in Figure 6.

**Figure 7:**
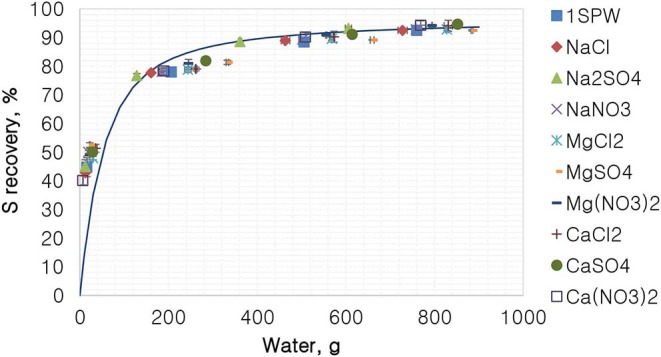
The %S recovery vs. water for all single salts and synthetic plant water.

Figure 7 shows the %S recovery as a function of the amount of water recovered for all tested single salts. According to Figure 7, all tested single salt solutions and synthetic plant water generally followed the same trend of a first-order model. It is shown that there was no difference in the recovery of total sulfides per gram of water regardless of the inorganic electrolyte solution used.

**Figure 8:**
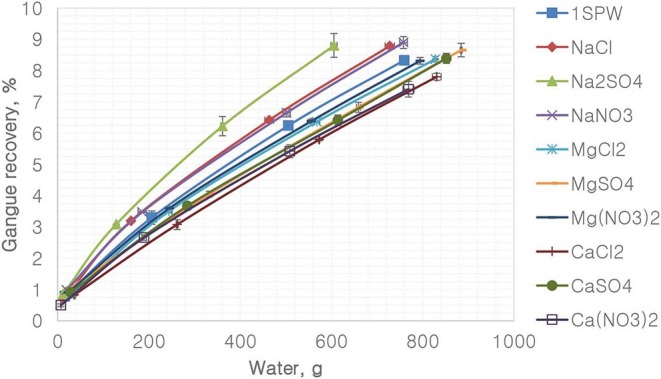
Gangue recovery vs. water for all single salts and synthetic plant water.

Figure 8 depicts the recovery of gangue per gram of water for the various single salts and the synthetic plant water. It is evident that all single salt solutions containing the monovalent Na^+^ resulted in higher gangue recovery per gram of water compared to that of the synthetic plant water and the single salt solutions containing divalent cations (Ca^2+^ and Mg^2+^). Ca^2+^- and Mg^2+^-containing single salt solutions resulted in lower gangue per gram of water compared to that of synthetic plant water. Furthermore, for the Na^+^ single salts, gangue recovery per gram of water was higher in the SO_4_^2−^ solution compared to the Cl^−^- and ${\text{NO}}_{3}^{-}$-containing solutions. Moreover, of the divalent cation-containing single salts, Ca^2+^ resulted in lower gangue recovery per gram of water compared to Mg^2+^.

### The Effect of Single Salts on the Coagulation of the Selected Merensky Ore

**Figure 9:**
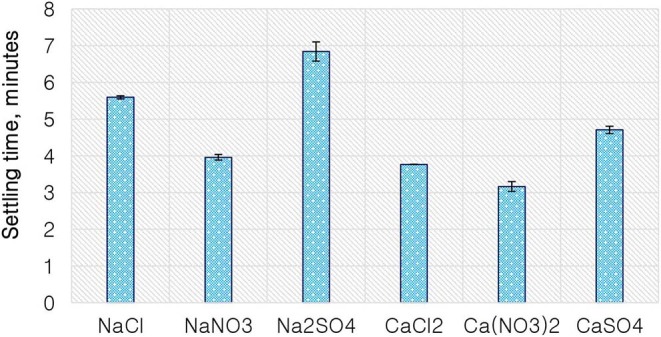
Merensky ore solids settling time as a function of the single salts.

Figure 9 shows the settling time of solids for a Merensky ore for various single salts as a proxy to cationic and anionic effect on coagulation. The Na^+^-containing solutions resulted in longer settling times compared to the Ca^2+^-containing solutions for every corresponding anion, suggesting a cation effect on coagulation with the divalent cation having a stronger coagulation effect compared to the monovalent cation. Moreover, for a fixed cation, SO_4_^2−^ resulted in the longest settling time followed by Cl^−^, while the ${\text{NO}}_{3}^{-}$ anions resulted in the shortest settling time.

### The Effect of Single Salts on the Zeta Potential of Talc

**Figure 10:**
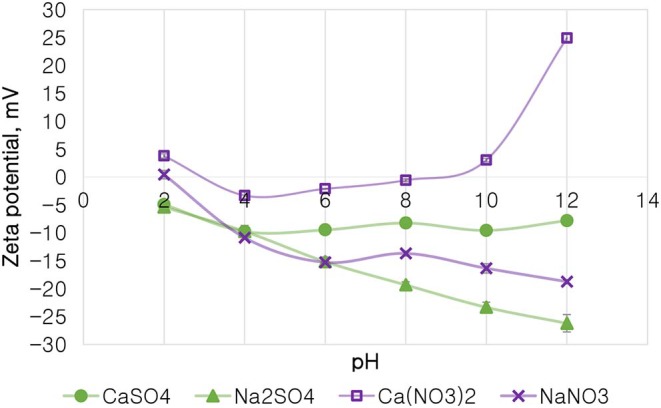
The zeta potential of talc in single salt solutions of 0.0213 mol dm^−3^ ionic strength as a function of pH.

Figure 10 depicts the zeta potential of talc as a function of pH in various single salt solutions in order to investigate whether there are any ion (anion and cation)-specific effects on the zeta potential of talc. Firstly, it is evident that the monovalent Na^+^ resulted in a highly negative zeta potential of talc across the studied pH range compared to solutions containing the divalent Ca^2+^. Secondly, for both cations, ${\text{NO}}_{3}^{-}$ resulted in a less negative zeta potential compared to SO_4_^2−^ across the studied pH range. Generally at pH > 4, Ca^2+^-containing single salt solutions resulted in an increase in the potential of talc with increasing pH, to an extent that the zeta potential of talc became positive after pH 8 for Ca(NO_3_)_2_. However, an increase in pH resulted in a further decrease in the potential of talc in Na^+^-containing solutions. A point of zero potential for talc is seen at pH 2 in NaNO_3_, while two points of zero potential for talc are seen with Ca(NO_3_)_2_ at pH 3 and pH 9. It is worth noting that at pH 4 the two SO_4_^2−^-containing solutions resulted in similar talc potentials.

### The Speciation of Selected Single Salt Solutions

**Figure 11:**
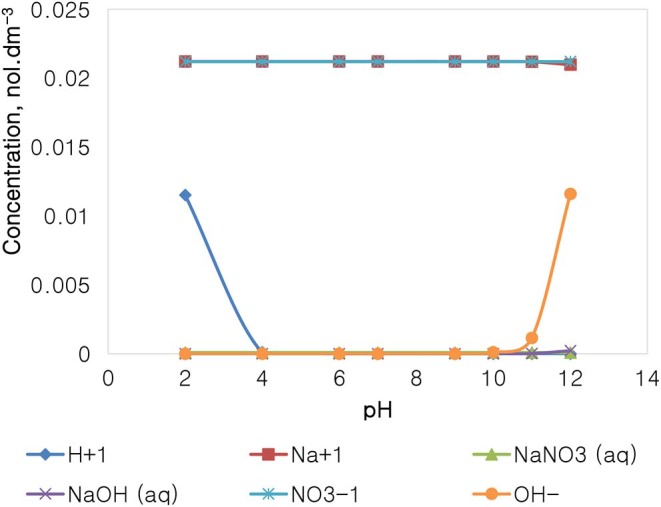
NaNO_3_ speciation for a solution of 0.0213 mol dm^−3^ ionic strength.

Figure 11 depicts the speciation of a NaNO_3_ solution, with an ionic strength of 0.0213 mol dm^−3^, generated through the Visual MINTEQ 3.1 software. The speciation of NaNO_3_ shows that at pH 4 and below, the dominant species are Na^+^, ${\text{NO}}_{3}^{-}$, and H^+^, with all of the other species existing at comparably insignificant concentrations. At the pH range of 4–10, only Na^+^ and ${\text{NO}}_{3}^{-}$ are dominant and present in solution. Beyond pH 10, the concentration of OH^−^ rises together with the apparent formation of NaOH(aq) and NaNO_3_(aq).

**Figure 12:**
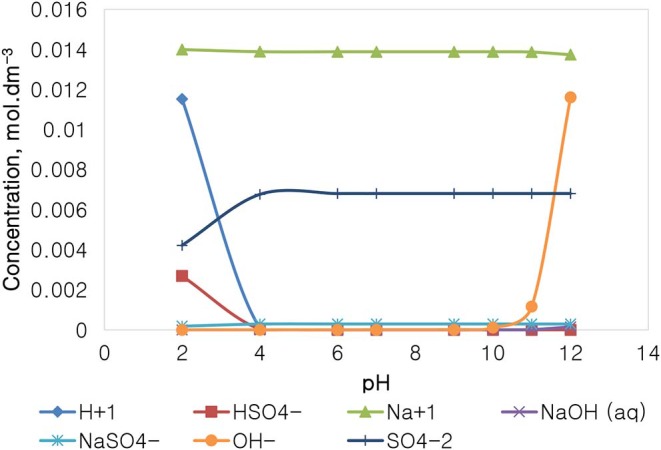
Na_2_SO_4_ speciation for a solution of 0.0213 mol dm^−3^ ionic strength.

Figure 12 depicts the speciation of a Na_2_SO_4_ solution, with an ionic strength of 0.0213 mol dm^−3^, generated through Visual MINTEQ 3.1 software. The speciation of Na_2_SO_4_ shows that below pH 4, contrary to the NaNO_3_ speciation, the dominant species are Na^+^, H^+^, and SO_4_^2−^, HSO${}_{4}^{-}$ as well as NaSO_4_. The concentration of these species remains fairly constant in the pH 4–10 range, except for HSO${}_{4}^{-}$ and H^+^ which become virtually insignificant, while the concentration of OH^−^ rises together with an appearance of NaOH(aq) and NaSO${}_{4}^{-}$ when the pH is increased beyond pH 10. It is important to note that the specific Na^+^ concentration in Na_2_SO_4_ is roughly 0.014 mol dm^−3^, relatively lower than that of 0.021 mol dm^−3^ in NaNO_3_ as reported in Figure 11, although both single salts have a fixed similar ionic strength of 0.021 mol dm^−3^. The ${\text{NO}}_{3}^{-}$ ion in NaNO_3_ also has a higher concentration compared to the concentration of SO_4_^2−^ seen in Na_2_SO_4_.

**Figure 13:**
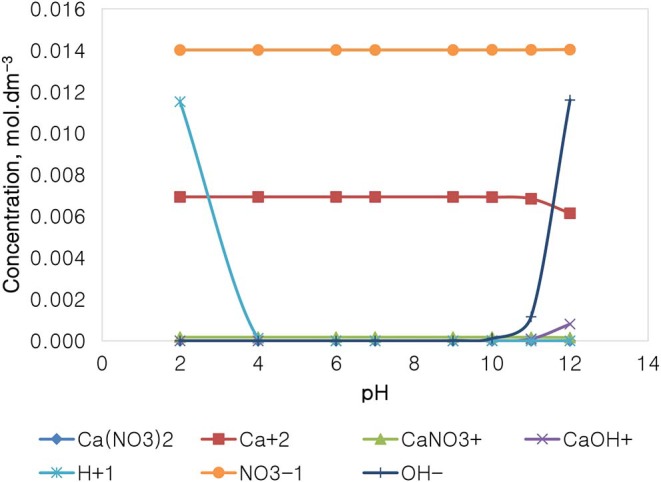
Ca(NO_3_)_2_ speciation for a solution of 0.0213 mol dm^−3^ ionic strength.

Figure 13 depicts the speciation of a Ca(NO_3_)_2_ solution, with an ionic strength of 0.0213 mol dm^−3^, generated through the Visual MINTEQ 3.1. The speciation of Ca(NO_3_)_2_ shows that at pH 4 and below, the dominant species are Ca^2+^, ${\text{NO}}_{3}^{-}$, and H^+^, with all of the other species existing at comparably insignificant concentrations. At the pH range of 4–10, only Ca^2+^ and ${\text{NO}}_{3}^{-}$ are dominant and present in the solution, with a concentration of 0.014 mol dm^−3^ for the ${\text{NO}}_{3}^{-}$ anions compared to that of the other species. It is also shown that above pH 10, the concentration of OH^−^ rises; however, that concentration is still relatively lower than that of the nitrate anions.

**Figure 14:**
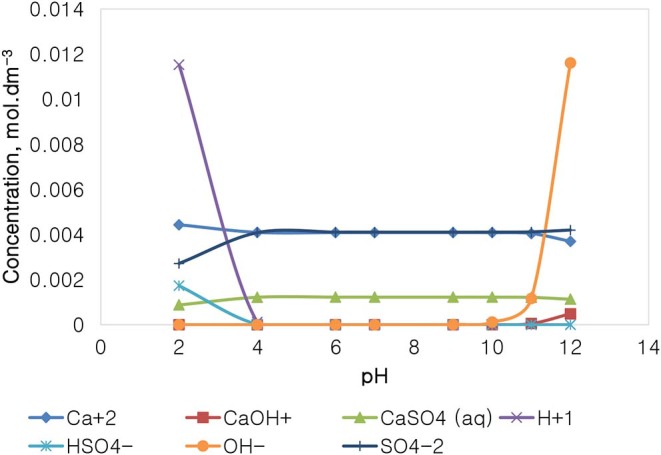
CaSO_4_ speciation for a solution of 0.0213 mol dm^−3^ ionic strength.

Figure 14 depicts the speciation of a CaSO_4_ solution, with an ionic strength of 0.0213 mol dm^−3^, generated through the Visual MINTEQ 3.1. The speciation of Na_2_SO_4_ shows that below pH 4, contrary to the NaNO_3_ speciation and similarly with that of Na_2_SO_4_, the dominant species are Ca^2+^, H^+^, and SO_4_^2−^, HSO${}_{4}^{-}$, and CaSO_4_. The concentration of these species remains fairly constant in the pH 4–10, while H^+^ becomes obsolete. The concentration of OH^−^ and of CaOH^+^ increases with an increase in the solution alkalinity, while the concentration of Ca^2+^ decreases with an increase in pH beyond pH 10. It is worth noting that the specific Ca^2+^ concentration in CaSO_4_ is roughly 0.004 mol dm^−3^, which is relatively lower than that of 0.014 mol dm^−3^ in Ca(NO_3_)_2_ as reported in Figure 12, although both single salts have a fixed similar ionic strength of 0.0213 mol dm^−3^. ${\text{NO}}_{3}^{-}$ is also present in higher concentrations (0.062 mol dm^−3^) compared to SO_4_^2−^ (0.003 mol dm^−3^).

## Discussion

Figures 1, 2 showed no evidence of specific ion effects on sulfide recoveries for the tested ore. Also, Figure 5 shows that the recovery of sulfides per gram of water did not change with ion type, indicating that specific ion effects on froth stability (as measured by the recovery of water) had little or no impact on the behavior of sulfides. However, specific ion effects were observed on the behavior of gangue in that Ca(NO_3_)_2_ and CaCl_2_ resulted in the lowest total amount of gangue reporting to the concentrate compared to those of all the other single salts. Figure 3 shows that NaNO_3_, Na_2_SO_4_, and NaCl resulted in the highest total recovery of gangue, while solutions containing Mg^2+^ were comparable to 1SPW. Figure 8 shows that the monovalent Na^+^ solutions resulted in the highest gangue recovery per gram of water compared to the divalent Ca^2+^ and Mg^2+^; this behavior is attributed to the effects on froth stability and the resulting impact on entrainment (Manono et al., 2018a). This behavior could imply that Na^+^ resulted in an activation of gangue, hence the higher gangue recovery per gram of water.

The specific ion effects seen on the behavior of gangue led to Ca(NO_3_)_2_ and CaCl_2_ resulting in the highest sulfide grades, whereas all the Na^+^ salts resulted in the lowest sulfide grades compared to the Ca^2+^ and Mg^2+^ single salt solutions. Thus, it can be said that the divalent Ca^2+^ and Mg^2+^ depressed the gangue more effectively. It was also apparent that in all cations (Ca^2+^, Mg^2+^, and Na^+^), solutions containing ${\text{NO}}_{3}^{-}$ depressed the gangue more readily compared to solutions containing Cl^−^ and SO_4_^2−^. This effect was stronger in the presence of Ca^2+^ compared to when a combination of Na^+^ and ${\text{NO}}_{3}^{-}$ was present in the solution.

Findings from the flotation results gave rise to the need to conduct further investigative work into the mechanism of the gangue depression effects seen, hence the determination of specific ion effects on the coagulation and the surface charge of talc. Talc was selected as a proxy for the naturally floatable gangue minerals needing to be depressed during the flotation of base metal sulfides-PGM-bearing ores. Single salts which showed the greatest impact on gangue depression and sulfide grades were selected for further investigative test work. Figure 9 shows that the divalent Ca^2+^ resulted in the shortest settling time compared to the monovalent Na^+^. This indicates that talc particles coagulated far easier in solutions containing Ca^2+^ compared to those in Na^+^-containing solutions. This trend is similar to the observations made in an earlier study by Manono et al. (2019) which showed enhanced coagulation and depression of talc in CaCl_2_ and MgCl_2_ compared to those in NaCl. Also, the solutions containing ${\text{NO}}_{3}^{-}$ resulted in a shorter settling time compared to those containing Cl^−^ and SO_4_^2−^. This implies that the ${\text{NO}}_{3}^{-}$-containing solutions resulted in increased coagulation of talc particles compared to the solutions containing Cl^−^ and SO_4_^2−^. These results are in line with the lower gangue recovery in the solutions containing Ca^2+^ and ${\text{NO}}_{3}^{-}$ compared to those in the solutions containing Na^+^, Cl^−^, and SO_4_^2−^ as seen in Figure 3.

On the basis of the gangue recovery and the coagulation trends seen in Figures 8, 9, it can be said that there exists specific ion effects on the behavior of gangue in that specific ions such as Ca^2+^ resulted in slightly more pronounced effects. These effects emanate from the passivation of gangue mineral particle surfaces due to the presence of species such as Ca^2+^, CaOH^+^, and CaNO_3_+ as shown in Figures 11–14. It is evident that beyond pH 9, oxyhydroxo species and metal complexes become dominant; these alter the surface chemistry of the mineral particles. These oxyhydroxo species are known to impart a more hydrophilic nature on the naturally hydrophobic gangue and thus cause a greater depression of gangue. Zeta potential measurements for talc were taken in various single salts of monovalent and divalent cations to probe the effect of inorganic electrolytes on the particle surface charge. Figure 10 shows that the zeta potential of talc was less negative in Ca^2+^ compared to that in Na^+^. These results correlate well with the greater depression and coagulation of gangue in Ca^2+^ compared to those in Na^+^ as shown in Figures 3, 9. This implies that the non-sulfide gangue mineral particles were more passivated by the presence of the divalent Ca^2+^ and its resulting oxyhydroxo species in solution such that the surface of the gangue mineral particles would become more hydrophilic. The resulting hydrophilicity would in turn enhance the coagulation of particles and thereby enhance their depression. A similar finding was observed for the anions which resulted in greater depression and coagulation of gangue in that the ${\text{NO}}_{3}^{-}$-containing solutions in all cation types resulted in a less negative zeta potential, particularly at the natural flotation pH 9, compared to that of the SO_4_^2−^-containing solutions. Furthermore, the zeta potential results as a function of pH correlate well with the speciation diagrams shown in Figures 11–14 in that the dominant oxyhydroxo species seen at pH > 9 are said to passivate mineral surfaces and thereby induce their hydrophilic nature, creating an environment conducive for gangue mineral depression (Laskowski et al., 2007; Ikumapayi et al., 2012; Feng et al., 2013). The coagulation findings of this work are in agreement with those of Dishon et al. (2009) who found that in highly concentrated electrolyte solutions, the adsorption of cations onto the mineral surface changed the surface charge of the particles and resulted in strong attractive forces between particles which consequently formed hydrophilic agglomerates. Similar to what has been shown in this present work on the depression of gangue, an investigation into the role of Ca^2+^ ions on the surface properties of molybdenite in copper porphyries by Li et al. (2015) showed that the floatability of fine molybdenite particles decreased significantly when Ca^2+^ ions and silica coexisted in the flotation pulp. Raghavan and Hsu (1984) relate this phenomenon to the adsorption of Ca^2+^ ions on mineral particles, reducing the magnitude of the negative surface charge and therefore causing the coagulation of mineral particles. As recorded in literature, it is worth mentioning that zeta potential changes also occur as a result of the influence of adsorption of ions onto the mineral surface (Manono et al., 2018b; Michaux et al., 2018). The adsorption of ions on the mineral surface and the resulting change in zeta potential are said to occur either by electrostatic attraction, chemisorption, or chemical reaction. The findings of this work confirm Gaudin (1932) and Gaudin and Charles (1953) who proposed that changes in the potential determining the ion concentration in the solution can cause a reduction in the zeta potential. Furthermore, the resulting correlation between the coagulation and zeta potential trends seen in this work confirms the proposal of a qualitative parallel between coagulation and zeta potential made by Fuerstenau and Mishra (1980) and Fuerstenau et al. (1988).

It is thus evident that the presence of divalent Ca^2+^ and the resulting oxyhydroxo species in process water causes an adsorption of these inorganic electrolytes onto the mineral surface. These consequently reduce the negative surface charge of the mineral particle as shown by the more positive zeta potential and passivated mineral surface in Figure 10. This in turn leads to enhanced coagulation and depression of gangue.

### Graphical Summary of Findings

From Graphical Abstract, it is therefore proposed that zeta potential measurements and coagulation tests can be used as a tool to test for effects that specific process water constituents will have on the surface chemistry response and floatability of naturally hydrophobic gangue minerals during the flotation of Cu-Ni-PGM ores. Knowledge of such gangue mineral surface chemistry responses to water quality changes may prove beneficial in determining the desired water quality and reagent regimes for uninterrupted flotation performance.

**GRAPHICAL ABSTRACT:**
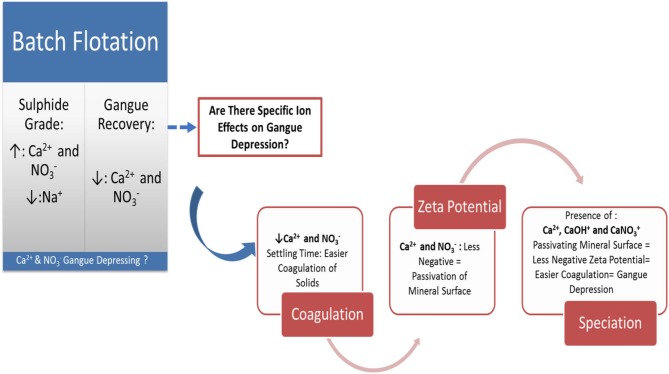
A schematic summary of the research findings presented in this paper.

## Conclusions

The findings of this work have shown that the monovalent Na^+^ did not promote coagulation. However, in Ca^2+^, coagulation was favored as less gangue was recovered into the concentrate, indicating an increased gangue depression in the presence of Ca^2+^ compared to Na^+^. This trend was more pronounced when Ca^2+^ was in combination with ${\text{NO}}_{3}^{-}$. The zeta potential results showed a less negative zeta potential of talc with Ca^2+^ compared to that with Na^+^, showing a greater passivation of the mineral surface in Ca^2+^-containing single salt solutions. The greater depression in ${\text{NO}}_{3}^{-}$ was confirmed by the less negative zeta potential, the lower gangue recoveries, and the shorter settling time. Thus, the key findings of this work are:

- Both ${\text{NO}}_{3}^{-}$ and Ca^2+^ increased the depression of gangue as shown by the lower gangue recoveries.
- The concentrate grades were greater in Ca^2+^ compared to those in Na^+^.
- Coagulation and zeta potential tests indicated that Ca^2+^ and ${\text{NO}}_{3}^{-}$ would mostly likely create a pulp chemistry environment that promotes gangue depression.
- The presence of Ca^2+^ in flotation process waters could be beneficial for the depression of floatable gangue.

This work has also shown that the depressive action of inorganic electrolytes was more pronounced on gangue, whereas little or no depression action was seen on the valuable sulfides as total sulfide recoveries were generally the same for this specific PGM ore.

## Data Availability Statement

All datasets generated for this study are included in the article/supplementary material.

## Author Contributions

All authors listed have made a substantial, direct and intellectual contribution to the work, and approved it for publication. MM conceptualized the work, carried out the research experiments, analyzed the experimental data, wrote the manuscript while KC and JW assisted with conceptualisation, advised on the experimental test work and data analyses, proofread and edited the manuscript.

### Conflict of Interest

The authors declare that the research was conducted in the absence of any commercial or financial relationships that could be construed as a potential conflict of interest.
